# Sex Differences in Excitatory and Inhibitory Function in the Primary Somatosensory Cortex during the Early Follicular Phase: A Preliminary Study

**DOI:** 10.3390/brainsci13050761

**Published:** 2023-05-05

**Authors:** Sayaka Anazawa, Koya Yamashiro, Taiki Makibuchi, Koyuki Ikarashi, Tomomi Fujimoto, Genta Ochi, Daisuke Sato

**Affiliations:** 1 Field of Health and Sports, Graduate School of Niigata University of Health and Welfare, 1398 Shimami-cho, Kita-Ku, Niigata 950-3198, Japan; wtm22006@nuhw.ac.jp (S.A.); wtm22005@nuhw.ac.jp (T.M.); 2 Department of Health and Sports, Niigata University of Health and Welfare, 1398 Shimami-cho, Kita-Ku, Niigata 950-3198, Japan; koyuki-ikarashi@nuhw.ac.jp (K.I.); fujimoto@nuhw.ac.jp (T.F.); ochi@nuhw.ac.jp (G.O.); daisuke@nuhw.ac.jp (D.S.); 3 Institute for Human Movement and Medical Sciences, 1398 Shimami-cho, Kita-Ku, Niigata 950-3198, Japan

**Keywords:** primary somatosensory cortex (S1), somatosensory evoked potentials, paired-pulse inhibition (PPI), sex differences

## Abstract

Background and objectives: We examined sex differences in the excitatory and inhibitory functions of the primary somatosensory cortex (S1) between males and females during the early follicular phase, when estradiol hormones are unaffected. Methods: Fifty participants (25 males and 25 females) underwent measurement of somatosensory evoked potentials (SEPs) and paired-pulse inhibition (PPI) in the S1; SEPs and PPI were elicited by constant current square-wave pulses (0.2 ms duration) delivered to the right median nerve by electrical stimulation. Paired-pulse stimulation occurred at 30- and 100-ms interstimulus intervals. Participants were randomly presented with 1500 (500 stimuli each) single- and paired-pulse stimuli at 2 Hz. Results: The N20 amplitude was significantly larger in female subjects than in male subjects, and the PPI-30 ms was significantly potentiated in female subjects compared to that in male subjects. Conclusions: The excitatory and inhibitory functions in S1 differ between male and female subjects, at least during the early follicular phase.

## 1. Introduction

The gonadal hormones estradiol and progesterone can alter human brain function and structure and are believed to contribute to sex differences in brain structure and function. For example, Pletzer et al. (2018) [1] reported estradiol-dependent preovulatory increases in bilateral hippocampal gray matter mass and progesterone-dependent postovulatory increases in the gray matter of the right basal ganglia using magnetic resonance imaging.

The functional balance between neural excitation and inhibition likely varies during the estrous cycles in adult rats [2] and humans [3,4]. Therefore, somatosensory evoked potentials (SEPs) and paired-pulse inhibition (PPI) are used to evaluate human excitatory and inhibitory neural functions. For example, when the median nerve is stimulated at the wrist joint, a component of SEP called N20 reflects summated excitatory postsynaptic potentials in primary somatosensory cortex (S1) area 3b pyramidal neurons at basal dendrites through specific thalamocortical afferents. This event is considered an indicator of local cortical excitability [5,6,7]; however, such events are uninfluenced by the cognitive state or attention [8,9]. Conversely, PPI measures inhibitory function in S1 [10,11,12].

PPI describes the outcome of stimulation with very short interstimulus intervals (ISI). Compared to initial cortical responses, responses to the second stimulus are significantly inhibited. PPI is quantified by calculating the ratio of the amplitude of the second response divided by the amplitude of the first response. Small amplitude ratios indicate potent inhibition, whereas large ratios indicate little inhibition (and enhanced excitation) [4]. This indicator is closely associated with the temporal discrimination threshold [11,12], aging [10,13], and motor learning [14].

Schloemer et al. (2020) [4] found estradiol to disinhibit S1 and the primary visual cortex in luteal phases compared to follicular phases in female subjects. According to Ikarashi et al. (2020) [3], compared to the follicular phase, PPI tended toward disinhibition during the ovulatory phase when estradiol concentrations peaked. These findings further our understanding of the disinhibitory effects on inhibitory function in S1. In addition, PPI was more strongly potentiated in the follicular phase in female subjects than in male subjects, but no difference was detected during the luteal phase. Thus, estradiol may influence sex differences in the human inhibitory function of S1, as recorded by PPI.

Sex differences also are evaluated by SEPs [15,16] and somatosensory evoked fields (SEFs) [17] in S1. Two studies have reported sex differences in SEPs, while another found no such differences in SEFs. However, because these studies did not consider menstrual cycle effects, the inconsistent results could be attributed to estradiol-induced S1 excitation.

A recent study that controlled for hormonal effects found that the peak-to-peak of N20 and P25 did not differ between male and female subjects between the follicular and luteal phases [4]. Recently, Edama et al. (2022) [18] showed that estradiol and progesterone levels were similar in males and females during the early follicular phase.

Therefore, this study aimed to identify potential sex differences in excitatory and inhibitory function in S1 using SEPs during the early follicular phase, when the estradiol and progesterone hormones are unaffected. We hypothesized that the N20 amplitude would be similar between male and female subjects, but PPI would be potentiated with no or minor effects of estradiol during the early follicular phase.

## 2. Materials and Methods

### 2.1. Participants

The study’s participants were 50 university students (25 males and 25 females) with no history of neurological or psychiatric disorders. This sample size was larger than the minimum of 24 needed for 80% power and a significance level of 5% based on a medium effect size of 0.60. The mean age of the 25 male subjects was 20.6 ± 1.4 years, and their mean height was 172.4 ± 5.4 cm. The mean age of the 25 female subjects was 21 ± 1.8 years, and their mean height was 160.5 ± 6.7 cm. Additionally, the participants were not taking prescription medications or using hormonal contraception. Written informed consent was obtained from all participants following an explanation of the study’s objectives and methodology. All the female participants underwent the experimental procedure during their follicular phase (menstruation days 2–4), when the effect of the sex steroid hormones was attenuated. A previous study reported that estradiol and progesterone levels in males and females did not differ significantly during only the early follicular phase [18]. The study was conducted following the Declaration of Helsinki and was approved by the Ethics Committee of Niigata University of Health and Welfare, Niigata, Japan (approval number: 18915).

### 2.2. SEP and PPI Recording

SEPs and PPI were measured using a SynAmps amplifier system controlled by the Scan 4.3 software (Neuroscan, El Paso, TX, USA) and connected to a 32-electrode Neuroscan Quickcap™ with electrodes distributed according to the international 10–20 system [12]. The active electrode was placed at C3′ (2 cm posterior of C3), the reference electrode over the mid-front (Fz) position, and a ground electrode at the pre-frontal Fp2 position. Electroencephalographic (EEG) signals were recorded at a sampling rate of 1000 Hz and high-pass filtered at 3 Hz. Filtered recordings from 20 ms before to 200 ms after the SEP or PPI trigger stimulus were saved. Records exceeding ±100 μV in peak amplitude were rejected, and the remaining records averaged within individual participants and across participants within groups (“grand averages”). SEPs and PPI were elicited by constant current square-wave pulses (0.2-ms duration) delivered to the right median nerve using an electrical stimulator connected to a surface bar electrode oriented with the proximal cathode. Following previous studies, the stimulus intensity was set to a level slightly above the motor threshold [19,20]. Paired-pulse stimuli were delivered at 30- and 100-ms intervals. All participants were randomly presented with 1500 (500 stimuli each) single stimuli and stimulus pairs at 2 Hz using a pulse control system (Pulse Timer II; Medical Try System, Tokyo, Japan) and were asked to ignore the stimulated hand.

### 2.3. Analyses

Peak amplitudes of the single-pulse SEPs (sp-SEPs) N20 and P25 were measured relative to the pre-stimulus baseline at the C3′ electrode. For the measurement of paired-pulse inhibition, the amplitude of the second SEP pair (SEP2) was obtained by subtracting the mean individual sp-SEP waveform and then calculating the SEP2/SEP1 amplitude ratio [10,12].

The Shapiro–Wilk test was performed to confirm distribution normality before the statistical analysis using GraphPad Prism v 9.1.0 (San Diego, CA, USA). Normally distributed data were analyzed using Welch’s *t*-test; Mann–Whitney’s test was used for non-normally distributed data. Between-group differences in amplitudes of N20, P25, and PPI ratios were indicated by *p*-values < 0.05 for all tests. Statistical results are presented as means and standard deviations when Welch’s test was used and medians when the Mann–Whitney test was used.

## 3. Results

### 3.1. Single-Pulse SEP Amplitudes of N20 and P25

**Figure 1:**
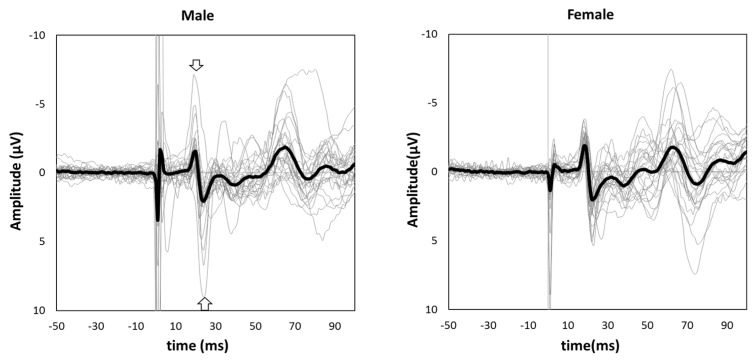
Grand-averaged somatosensory evoked potential (SEP) waveforms in male (**left panel**) and female subjects (**right panel**). The thick black lines show grand-averaged waveforms, and the thin gray lines show individual waveforms. The negative peak indicated by an arrow is N20, and the positive peak indicated by an arrow is P25.

**Figure 2:**
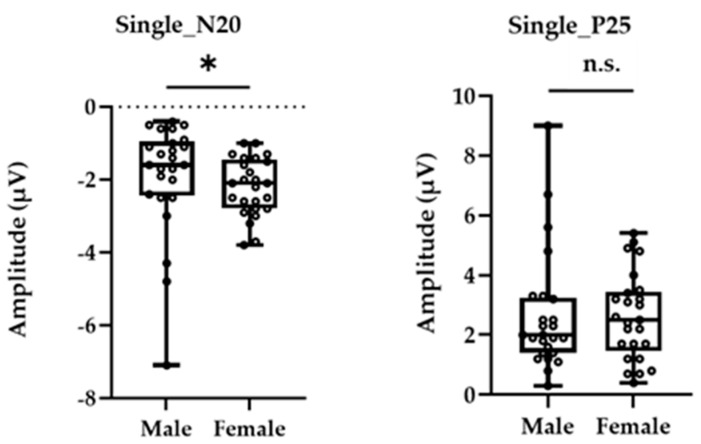
The peak amplitudes of N20 (**left panel**) and P25 (**right panel**) were extracted for each participant. The asterisk (*) and n.s. indicated *p* < 0.05 and no significant differences, respectively.

Figure 1 shows the grand-averaged waveform for single-pulse median nerve stimulation at the C3′ electrode position for males and females. Mann–Whitney’s test was performed for the N20 and P25 amplitudes due to the non-normal distributions at these positions. The amplitude of the sp-SEP, N20, was significantly larger in females than in males (−2.10 [−2.80 to −1.45] vs. −1.60 [−2.45 to −0.95], *p* = 0.037, Figure 2). There were no significant between-group differences observed for P25 amplitude (2.50 [1.45–3.45] vs. 2.00 [1.40–3.25], *p* = 0.613, Figure 2).

### 3.2. PPI-30 ms and 100 ms

**Figure 3:**
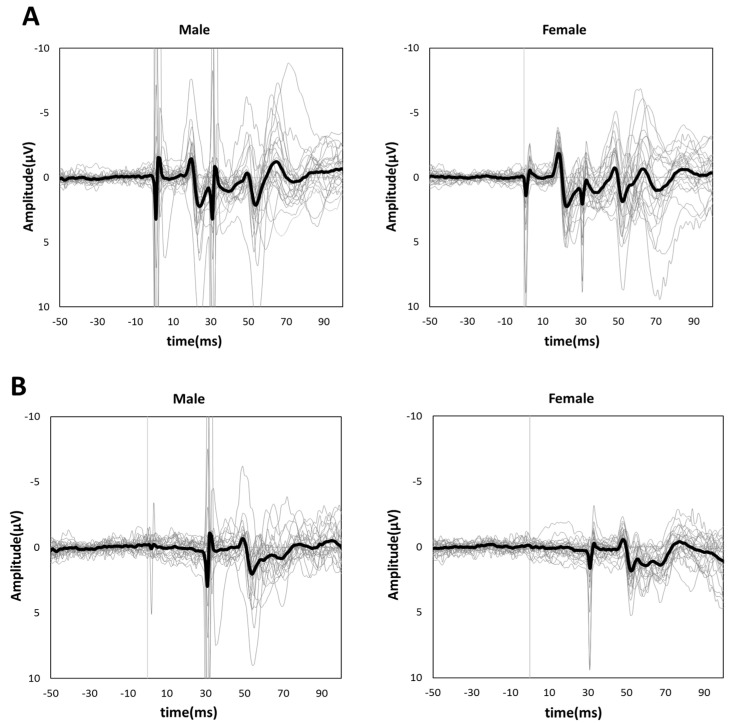
Grand-averaged (**A**) PPI-30 ms and (**B**) subtracted waveforms in male (**left panel**) and female subjects (**right panel**). The thick black lines show grand-averaged waveforms for males and females, respectively. The thin gray lines show individual waveforms.

**Figure 4:**
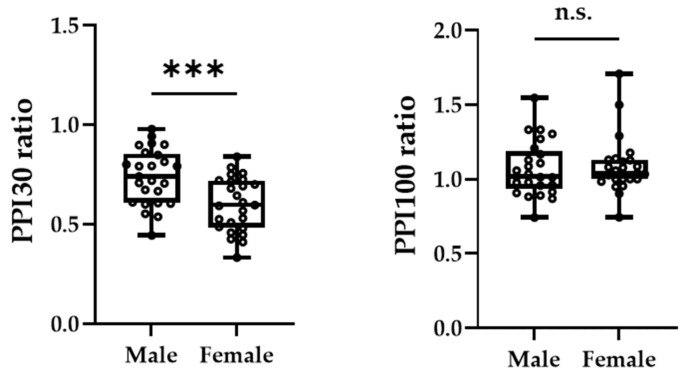
The peak amplitudes of PPI-30 ms ratio (**left panel**) and PPI-100 ms ratio (**right panel**) were extracted for each participant. The asterisk (***) and n.s. indicated *p* < 0.001 and no significant differences, respectively.

Figure 3A,B shows the grand-averaged waveforms for PPI-30 ms and subtracted at the C3′ electrode position for the male and female groups, respectively. An unpaired *t*-test was performed because it was confirmed to be normally distributed in PPI-30 ms. PPI-30 ms was more significantly inhibited in females than males (0.60 ± 0.14 vs. 0.74 ± 0.14, t = 3.61, *p* = 0.0007, Figure 4). Mann–Whitney’s test was performed for PPI-100 ms because of its non-normal distribution, which revealed no significant between-group differences (1.04 [1.00–1.13] vs. 1.02 [0.94–1.19], *p* = 0.61, Figure 4).

## 4. Discussion

We compared the SEPs and PPI in males and females to examine whether sex differences alter somatosensory excitatory and inhibitory function in S1 during the early follicular phase, when the estradiol hormones are unaffected. Our results showed increased N20 amplitudes and potentiated the inhibition effect of PPI-30 ms in females; no sex differences were noted for P25 amplitude and PPI-100 ms.

Very few studies have examined sex differences in humans’ short-latency SEPs (N20 and P25) [15,16,21]. Ikuta et al. (1982) [15] compared the SEPs of 100 young males and 100 females and found that, after median nerve stimulation, the amplitudes were significantly larger in females than in males, except for N20. Kakigi et al. (1991) [16] also compared SEPs in males and females and reported that N20 amplitudes were larger in females aged 20–39. The authors also reported larger SEP amplitudes in females than in males. Thus, age-specific sex differences in N20 amplitude may result from differences in brain maturation; however, the physiological significance of these differences could not be determined.

Huttunen et al. (1999) [17] measured SEFs (e.g., N20 m, P25 m, and P60 m) using magnetoencephalography (MEG) because SEFs are not significantly affected by cranial tissue conduction and, therefore, directly reflect neuronal activation. The researchers found no sex differences for N20 m, P35 m, and P60 m and concluded that the observed sex difference in S1 may be due to differences in volume conduction. Our N20 amplitude finding was consistent with Kakigi et al. (1991) [16]. If increased N20 amplitudes only existed as a product of cranial tissue conductivity, then the P25 amplitude would also be larger, as in a previous study [15]. Thus, sex differences in N20 and P25 amplitudes could reflect functional differences in males and females, at least in the early follicular phase. In addition, a recent study that controlled for hormonal effects found that the peak-to-peaks of N20 and P25 did not differ between males and females [4]. Therefore, the discrepancy in the present result might be due to the different hormonal effects between N20 and P25 amplitudes. In fact, the present study found a significant difference in N20, but not P25, amplitudes.

In the early follicular phase, when there were minimal differences in estradiol concentrations [18], PPI-30 ms was higher in female subjects than in male subjects, indicating sex differences in inhibitory neural activity. These results are consistent with previous studies [4]. However, in the preovulatory phase, with larger differences in estradiol concentrations, no sex differences were found [4], suggesting that sex differences in inhibitory neural activity in S1 occur primitively or because of other factors, but the influence of female hormones such as estradiol eliminates these differences. This is because estradiol reduces GABAergic inhibitory neural activity, effectively increasing the excitatory drive of pyramidal cells in rats [22]. Further research is required to investigate the mechanisms of sex differences in inhibitory neural activity in the early follicular phase.

Furthermore, the transient reduction of PPI-30 ms accompanied various sensory events. For example, Hoffken et al. (2007) [23] reported that the degree of improvement in two-point discrimination induced by tactile coactivation was correlated with a reduction in PPI-30 ms. Yamazaki et al. (2020) [12] also reported that PPI-30 ms was disinhibited by 50% VO_2_ peak exercise. Reductions in PPI-30 ms were non-significantly correlated with temporal discrimination. Since PPI was closely associated with sensorimotor function, females’ sensorimotor function in the early follicular phase may be inferior compared to that observed during the ovulatory phase or the luteal phase, or in males. Thus, excitatory and inhibitory functions in S1 differed in males and females during the early follicular phase. However, the major limitation of this study was that we only controlled the menstrual cycle phase, without measuring the blood or saliva levels of estrogen and progesterone. Future studies should control for the menstrual phase and measure estrogen and progesterone using blood or saliva tests.

## Data Availability

The data presented in this study are available on request from the corresponding author.
